# Microwave-Assisted Facile Synthesis, Anticancer Evaluation and Docking Study of *N*-((5-(Substituted methylene amino)-1,3,4-thiadiazol-2-yl)methyl) Benzamide Derivatives

**DOI:** 10.3390/molecules22060995

**Published:** 2017-06-15

**Authors:** Shailee V. Tiwari, Sumaiya Siddiqui, Julio A. Seijas, M. Pilar Vazquez-Tato, Aniket P. Sarkate, Deepak K. Lokwani, Anna Pratima G. Nikalje

**Affiliations:** 1 Y. B. Chavan College of Pharmacy, Dr. Rafiq Zakaria Campus, Rauza Bagh, Maharashtra, Aurangabad 431001, India; shailee2010@gmail.com (S.V.T.); sumaiyahsiddiqui@gmail.com (S.S.); dklokwani@gmail.com (D.K.L.); 2 Departamento de Química Orgánica, Facultad de Ciencias, Universidad of Santiago De Compostela, Alfonso X el Sabio, Lugo 27002, Spain; julioa.seijas@usc.es (J.A.S.); pilar.vazquez.tato@usc.es (M.P.V.-T.); 3 Department of Chemical Technology, Dr. Babasaheb Ambedkar Marathwada University, Maharashtra, Aurangabad 431004, India; aniketpharma1@gmail.com

**Keywords:** microwave-assisted synthesis, thiadiazoles, in vitro anticancer activity, molecular docking, ADMET

## Abstract

In the present work, 12 novel Schiff’s bases containing a thiadiazole scaffold and benzamide groups coupled through appropriate pharmacophore were synthesized. These moieties are associated with important biological properties. A facile, solvent-free synthesis of a series of novel **7**(**a**–**l**) *N*-((5-(substituted methylene amino)-1,3,4-thiadiazol-2-yl)methyl) benzamide was carried out under microwave irradiation. Structures of the synthesized compounds were confirmed by IR, NMR, mass spectral study and elemental analysis. All the synthesized hybrids were evaluated for their in vitro anticancer activity against a panel of four human cancer cell lines, viz. SK-MEL-2 (melanoma), HL-60 (leukemia), HeLa (cervical cancer), MCF-7 (breast cancer) and normal breast epithelial cell (MCF-10A) using 3-(4,5-dimethythiazol-2-yl)-2,5-diphenyl tetrazolium bromide (MTT) assay method. Most of the synthesized compounds exhibited promising anticancer activity, showed comparable GI_50_ values comparable to that of the standard drug Adriamycin. The compounds **7k**, **7l**, **7b**, and **7a** were found to be the most promising anticancer agents in this study. A molecular docking study was performed to predict the probable mechanism of action and computational study of the synthesized compounds **7**(**a**–**l**) was performed to predict absorption, distribution, metabolism, excretion and toxicity (ADMET) properties, by using QikProp v3.5 (Schrödinger LLC). The results showed the good oral drug-like behavior of the synthesized compounds **7**(**a**–**l**).

## 1. Introduction

Cancer is a disease in which cells grow and proliferate in an uncontrolled manner. Cancer evokes a high level of mortality regardless of the recent advances in the development of clinically authorized anticancer agents [1,2,3].

The development of resistance to chemotherapeutic agents and associated side effects are major obstacles to effectively treating cancer [3]. Thus, it is necessary to identify and develop new anticancer agents with improved efficacy and reduced side effects to complement the present chemotherapeutic strategies. Hence, further research towards the development of newer therapeutic drugs that more effectively combat cancer is needed.

One of the potential targets for new drugs is the matrix metalloproteinases (MMPs), a group of proteinases that have physiologic roles in degrading and remodeling the extracellular membrane. The MMPs are over-expressed in a variety of malignant tumor types, and their over-expression is associated with tumor aggressiveness and metastatic potential [4,5,6,7,8]. The specific alteration of the MMPs observed in malignant tissues and their participation in some of the major oncogenic mechanisms have fueled interest in the design and evaluation of MMP inhibitors as anticancer agents [9,10,11,12].

The class of organic compounds containing the azomethine (–HC=N–) group in their structure is called imine, or alternatively a Schiff base. Schiff bases, derived mostly from a variety of heterocyclic rings, were reported to possess a broad spectrum of pharmacological activities with a wide variety of biological properties, and development of new chemotherapeutic Schiff bases is now attracting the attention of medicinal chemists. They are known to exhibit a variety of potent biological activities.

Heterocyclic nucleus 1,3,4-thiadiazole constitutes an important class of compounds for new drug development because of its interesting biological properties, which include anticancer [13], antibacterial [14] and antifungal activity [15]. Thiadiazoles (e.g., 1,3,4-thiadiazoles) also exhibit a number of extremely interesting spectroscopic properties. One of the very interesting spectroscopic properties of thiadiazoles is the dual fluorescence effect [16,17,18,19]. In addition, 1,3,4-thiadiazole compounds are excellent ligands and a good skeleton for metallo-complexes [20]. Especially, the structure of ‘‘N–C–S’’ in 1,3,4-thiadiazole derivatives can work as the active center, chelate certain metal ions in vivo and show good tissue permeability. The synthesis of novel thiadiazole derivatives and investigation of their chemical and biological behavior have gained more importance in recent decades [21,22,23,24]. The 1,3,4-thiadozole compounds have been reported to inhibit MMPs [25]. Structure–activity relationship (SAR) studies have shown that thiadiazoles are selective for stromelysin-1(MMP-3) over gelatinase-A (MMP-2) with little or no affinity for collagenase (MMP-1) [25]. The dual roles that matrix metalloproteinases appear to play in cancer growth and metastasis [26,27] highlighted the need for studies that address a physical basis for selective inhibition of specific MMPs.

In the present work we planned to develop Schiff’s bases containing a thiadiazole ring and benzamide groups, because of the important biological properties associated with these groups. The coupling of these important moieties was achieved under microwave irradiation. Microwave-induced synthesis has various advantages over conventional synthesis, such as a highly accelerated reaction rate, reasonably good yields, a lesser solvent requirement, an eco-friendly method, a clean heating system and controlled reaction parameters [28].

All the synthesized hybrids were evaluated for their in vitro anticancer activity against a panel of four human cancer cell lines viz. against MCF-7 (human breast cancer cell line), HeLa (human cervical cancer cell line), SKMEL-2 (human melanoma cancer cell line) and HL-60 (human leukemia cancer cell line) by MTT assay using Adriamycin as the standard drug. A molecular docking study has also been performed to support the effective binding of the compound at the active site of the enzyme. Most of the synthesized compounds exhibited promising anticancer activity, with some compounds having GI_50_ values equivalent to that of the standard drug, Adriamycin. The compounds **7k**, **7l**, **7b** and **7a** were found to be the most promising anticancer agents in this study. All the synthesized compounds were screened for their in silico absorption, distribution, metabolism, excretion and toxicity (ADMET) study as well.

## 2. Result and Discussion

### 2.1. Chemistry

**Scheme 1:**
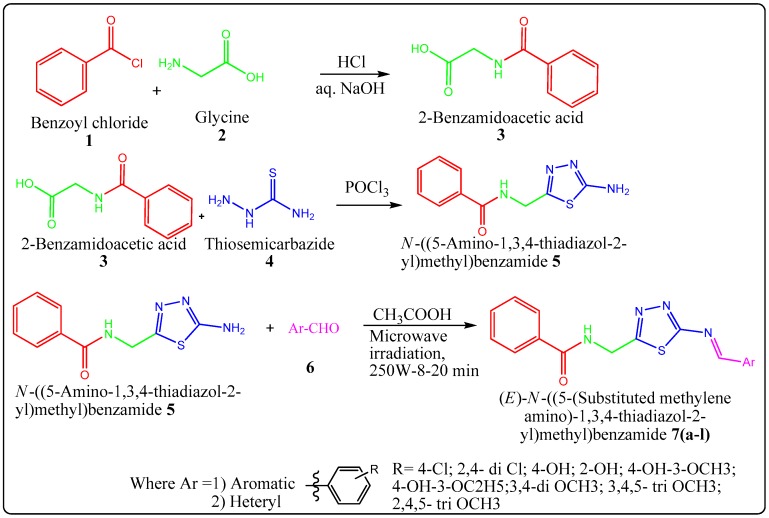
Synthesis of *N*-((5-(Substituted methylene amino)-1,3,4-thiadiazol-2-yl)methyl) benzamide derivatives **7**(**a**–**l**).

Herein, we report the facile synthesis of novel *N*-((5-(substituted methylene amino)-1,3,4-thiadiazol-2-yl)methyl) benzamide derivatives under microwave irradiation as shown in Scheme 1. The *N*-((5-(substituted methylene amino)-1,3,4-thiadiazol-2-yl)methyl) benzamide derivatives were synthesized from *N*-((5-amino-1,3,4-thiadiazol-2-yl)methyl) benzamide (0.01 mol) (**5**) and various suitable aldehydes (**6**) (0.01 mol) using conventional method and microwave method, as shown in Table 1. All the synthesized compounds were characterized by ^1^H-NMR, ^13^C-NMR, mass spectroscopy, IR and elemental analysis.

### 2.2. In Vitro Anticancer Activity

The synthesized compounds **7**(**a**–**l**) were evaluated for their in vitro anticancer activity against MCF-7 (human breast cancer cell line), HeLa (human cervical cancer cell line), SKMEL-2 (human melanoma cancer cell line) and HL-60 (human leukemia cancer cell line) cancer cell lines, as shown in Table 2. The most active compounds **7k**, **7l**, **7a** and **7b** were tested on non-tumorigenic breast epithelial cell lines (MCF-10A) to find out the selectivity towards the cancer cells. The GI_50_ values (the concentration required to achieve a growth inhibition of 50%) for the synthesized compounds were determined by MTT assay method. The in vitro anticancer evaluation results and GI_50_ values were listed in Table 2, and the well-known anticancer drug Adriamycin was used as positive control.

The results indicated that the compounds **7k**, **7l**, **7a** and **7b** exhibited significant cancer cell growth inhibition compared to reference standard Adriamycin against MCF-7, HeLa, SKMEL-2 and HL-60 cancer cell lines. From the in vitro anticancer activity results, it was observed that compound **7k**, which has a furan ring, was found to have the GI_50_ values 11.7 µM, 23.8 µM, 19.6 µM and 35.5 µM for MCF-7, HeLa, SKMEL-2 and HL-60 cancer cell lines, respectively. Compound **7l**, which has a thiophene ring, was found to have the GI_50_ values 19.0 µM, 28.8 µM, 22.0 µM and 29.9 µM for MCF-7, HeLa, SKMEL-2 and HL-60 cancer cell lines, respectively. Compound **7a** containing 4-Chlorophenyl substituent (MCF-7 GI_50_ 22.9 µM, HeLa GI_50_ 32.8 µM, SKMEL-2 GI_50_ 21.9 µM and HL-60 GI_50_ 21.7 µM) and compound **7b** 2,4 dichloro phenyl substituent (MCF-7 GI_50_ 28.7 µM, HeLa GI_50_ 39.0 µM, SKMEL-2 GI_50_ 22.9 µM and HL-60 GI_50_ 28.2 µM) were found to have good in vitro anticancer activity.

From the in vitro anticancer result data as shown in Table 2, the most active compounds **7k**, **7l**, **7a** and **7b** were tested on non-tumorigenic breast epithelial cell lines (MCF-10A) to find out the selectivity towards the cancer cells. Interestingly, compounds **7k**, **7l** and **7a** were found to be selective towards cancer cells, since they did not exhibit cytotoxicity (GI_50_ >100 μM) on MCF-10A cells. However, compound **7b** showed almost three times more selectivity for MCF-7 cancer cell lines in comparison to the MCF-10A normal breast epithelial cell lines.

Structure-activity relationship (SAR) studies for these compounds demonstrated that electron withdrawing groups such as chloro (**7a**, **7b**) exhibited good in vitro anticancer activity compared to electron donating groups. Compound **7c** with 4-hydroxyphenyl substituent (MCF-7 GI_50_ 32.4 µM, HeLa GI_50_ 41.1 µM, SKMEL-2 GI_50_ 27.5 µM and HL-60 GI_50_ 33.3 µM) was found to be more active than compound **7e** with 4-hydroxy-3-methoxyphenyl substituent (MCF-7 GI_50_ 35.2 µM, HeLa GI_50_ 46.8 µM, SKMEL-2 GI_50_ 28.1 µM and HL-60 GI_50_ 39.6 µM) and compound **7f** with 4-hydroxy-3-ethoxy phenyl substituent (MCF-7 GI_50_ 38.4 µM, HeLa GI_50_ 49.2 µM, SKMEL-2 GI_50_ 30.0 µM and HL-60 GI_50_ 37.5 µM). Replacement of the phenyl group in the parent compound by furan ring in **7k** and thiophene ring in **7l** showed a significant increase in the in vitro anticancer activity in comparison to the standard drug Adriamycin. From SAR, it can be considered that compounds containing electron-donating polar groups such as **7c**, **7d**, **7e**, **7f**, **7g**, **7h**, **7i** and **7j** are less active in comparison to the electron-withdrawing groups, such as **7a** and **7b**. It is also clear that the replacement of the phenyl ring with the furan ring (**7k**) and thiophene ring (**7l**) significantly increased the in vitro anticancer activity. Compounds having large, bulky di-substituted and tri-substituted groups on phenyl ring showed a decrease in in vitro anticancer activity (for example, **7e** and **7f**) than compounds having less bulky substituents on phenyl ring, such as **7a** and **7b**. Compounds **7k** and **7l** showed significantly good in vitro anticancer activity in comparison to the standard anticancer drug Adriamycin against MCF-7, HeLa, SKMEL-2 and HL-60 cancer cell lines and thus can be developed as anticancer agents in the future.

### 2.3. Molecular Docking

Unregulated or over-expressed matrix metalloproteinases (MMPs), including stromelysin, collagenase and gelatinase, were implicated in several pathological conditions, including arthritis and cancer. The 1,3,4-thiadozole compounds have been reported to inhibit selectively fibroblast stromelysin-1 [24,29]. In order to predict the binding affinity of our newly synthesized *N*-((5-(substituted methylene amino)-1,3,4-thiadiazol-2-yl)methyl) benzamide **7**(**a**–**l**) derivatives into the binding site of fibroblast stromelysin-1, we decided to carry out a molecular docking study. The docking results indicated that compounds were held in the active pocket by a combination of various hydrogen bonds and hydrophobic interactions with fibroblast stromelysin-1. The docking results revealed that the compound **7k** had the highest binding interaction with fibroblast stromelysin-1, with a docking score of −7.56, as shown in Table 3.

On the basis of the docking results, it was found that compounds **7k**, **7l**, **7a** and **7b** had good potential to inhibit fibroblast stromelysin-1, which will be the probable mechanism of these derivatives for their anticancer activity. The docking score of all the synthesized derivatives is shown in Table 3.

**Figure 1:**
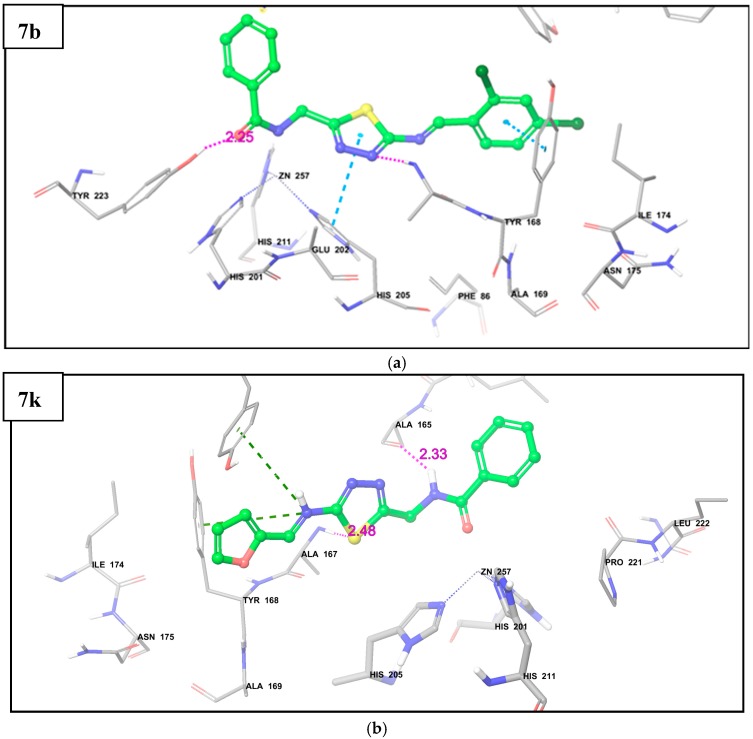
Docking pose of: (**a**) Compound **7b**; (**b**) Compound **7k** in the active pocket of Fibroblast stromelysin-1. The pink-colored dotted line indicates H-bond, the cyan-colored dotted line indicates pi-pi stacking interaction, and the green-colored dotted line indicates pi-pi cation interaction between ligand and enzyme.

From the docking analysis, it was observed that compound **7b** showed hydrogen bond interaction with amino acid residue Tyr223; while on the other hand, hydrogen bonding is seen with amino acid residues Ala165 and Ala167 in compound **7k** (Figure 1). The hydrophobic interactions such as pi-pi stacking and pi-pi cation between compounds and surrounding hydrophobic amino acid residues of enzymes helped to stabilize the complex structure and thus enhanced the binding affinity of compounds towards fibroblast stromelysin-1 (Figure 1). It was also seen that the –CONH group of both compounds **7b** and **7k** makes a metal coordination with zinc ion present in the active site of the enzyme, which is a probable mechanism of inhibition of fibroblast stromelysin-1.

### 2.4. In Silico ADMET Prediction

The absorption, distribution, metabolism, excretion and toxicity (ADMET) play a critical role in helping to optimize the pharmacokinetic properties of new drugs, as it increases their success rate. Thus the prediction of the ADMET parameters prior to experimental studies is one of the most important aspects of both drug discovery and development of the drug molecule. The prediction of Lipinski’s rule-of-five was performed to indicate whether a chemical compound could be an orally active drug in humans. The prediction of ADMET properties was performed for all compounds and is shown in Table 4. It was observed that all the compounds exhibited a good predicted percentage of oral absorption (% ABS) ranging from 75–100%. The predicted aqueous solubility (Log S) (S in mol/L) and predicted partition coefficient (Log Po/w) value for all compounds was found within the range of −6.1 to −4.2 and 3.91–5.34 respectively, and thus is a probable reason for the predicted good percentage of oral absorption for all compounds. The efficiency and distribution of a drug may be affected by the degree to which it binds to the proteins present within the blood plasma. The log Khsa parameter is used for the prediction of binding to the human serum albumin. All compounds showed predicted log Khsa value ranges between −0.2 and 0.19, which indicated that a significant proportion of the compounds are likely to circulate freely in the blood stream and can reach the drug target sites.

Human ether-a-go-go related gene (HERG) encodes a potassium ion (K^+^) channel that is implicated in the fatal arrhythmia known as *torsade de pointes,* or the long QT syndrome [30]. The HERG K^+^ channel, which is best known for its contribution to the electrical activity of the heart that coordinates the heart’s beating, appears to be the molecular target responsible for the cardiac toxicity of a wide range of therapeutic drugs [31]. HERG has also been associated with modulating the functions of some cells of the nervous system, and with establishing and maintaining cancer-like features in leukemic cells [32]. Thus, HERG K^+^ channel blockers are potentially toxic and the predicted IC_50_ values often provide reasonable predictions for the cardiac toxicity of drugs in the early stages of drug discovery [33]. Thus, based on predicted log HERG values, none of the synthesized compounds **7**(**a**–**l**) were found to be toxic in nature. Similarly, it was also seen that none of the compounds have violated the Lipinski rule-of-five (M.W < 500, l Log Po/w<5, *n*-ON < 10, *n*-OHNH < 5). The overall ADMET prediction studies thus clearly suggested that all synthesized compounds have good pharmacokinetic properties and can be developed as good oral drugs in the future.

## 3. Materials and Methods

### 3.1. General Information

All the chemicals used for synthesis were of Merck, Sigma, Research lab, Qualigens make and Himedia. The reactions were carried out by conventional method and in synthetic microwave oven (Milestone micro synth). Melting points were determined in open capillaries using melting point apparatus and are uncorrected. All the reactions were performed in oven-dried glassware. Phosphorus oxychloride was used by distilling under reduced pressure. The synthetic protocol employed for the synthesis of *N*-((5-(substituted methylene amino)-1,3,4-thiadiazol-2-yl)methyl) benzamide derivatives **7**(**a**–**l**) is presented in Scheme 1. The purity of the synthesized compounds was checked by TLC, and melting points were determined in open capillary tubes and are uncorrected. The NMR spectra of the final titled compounds *N*-((5-(substituted methylene amino)-1,3,4-thiadiazol-2-yl)methyl) benzamide were recorded on Brucker Advance II (400 MHz), and Infrared (IR) spectra were recorded for the compounds on JASCO FTIR (PS 4000, JASCO, Tokyo, Japan) using KBr pallet. The mass spectra were recorded on a Waters Micro Mass (ZQ 2000 spectrometer, UK). Elemental analyses were done with a FLASHEA 112 Shimadzu’ analyzer (Mumbai, Maharashtra, India) and all analyses were consistent (within 0.4%) with theoretical values.

#### 3.1.1. Step I: General Process for Synthesis of 2-Benzamidoacetic Acid [34]

The first step involved dissolving 0.33 mol of 2-aminoacetic acid (**2**) in 10% NaOH solution contained in a conical flask. To this solution, 0.38 mol of benzoyl chloride (**1**) was added in five portions and shaken vigorously until the chloride reacted. The solution was transferred to a beaker containing crushed ice and dil. HCl was added until the solution was acidic to congo red paper. The resulting crystalline solid was collected and boiled with 10 mL of CCl_4_ for 10 min. The product was filtered and washed with CCl_4_. The solid product obtained was dried and recrystallized from ethanol. The melting point and yield were recorded. 

#### 3.1.2. Step II: General Procedure for Synthesis of *N*-((5-Amino-1,3,4-thiadiazol-2-yl)methyl) Benzamide [35]

Next, 2-benzamidoacetic acid (**3**) (0.05 mol) was refluxed with thiosemicarbazide (**4**) (0.05 mol) and phosphorus oxychloride (15 mL) for 1 h. The mixture was cooled and diluted with water (90 mL) and again refluxed for 4 h. Then the mixture was filtered, and the filtrate was basified with potassium hydroxide solution. The precipitate was filtered off and recrystallized from ethanol.

#### 3.1.3. Step III: General procedure for synthesis of (*E*)-*N*-((5-(substituted methyleneamino)-1,3,4-thiadiazol-2-yl)methyl) benzamide **7**(**a**–**l**)

A. Conventional method [36]

Equimolar quantities of *N*-((5-amino-1,3,4-thiadiazol-2-yl)methyl) benzamide (0.01 mol) (**5**) and various suitable aldehydes (**6**) (0.01 mol) were refluxed in the presence of glacial acetic acid (0.02 mol) in absolute ethanol (25 mL) for 3–8 h. The completion of the reaction was monitored by Thin Layer Chromatography (TLC). The reaction mixture was concentrated and cooled. The obtained solid was filtered and dried. The product was recrystallized from ethanol. The melting point and yield were recorded.

B. Microwave-assisted method [37]

Solvent-free synthesis of Schiff’s bases was achieved by cyclo addition of various suitable aldehydes (**6**) (0.01 mol) and *N*-((5-amino-1,3,4-thiadiazol-2-yl)methyl) benzamide (**5**) (0.01 mol) in the presence of a catalytic amount of glacial acetic acid under microwave irradiation at 250 W for 8–20 min, as shown in Scheme 1. The completion of the reaction was monitored by TLC. The synthesized products were recrystallized from ethanol. The same compounds were also synthesized using conventional approach. A comparative study in terms of yield and reaction period has been reported for both microwave-assisted and conventional methods. The conventional method required about 3–8 h, while the microwave-irradiation method required only 8–20 min, as shown in Table 1.

2-Benzamidoacetic acid **3**. Yield: 95%; m.p.: 187–190 °C;IR (KBr ν_max_ in cm^−1^): 3350.41 (NH), 3179.92 (OH), 2970.76 (C=H), 1810.26 (C=O of amide), 1725.01 (C=O of carboxylic acid); ^1^H-NMR (DMSO) δ ppm: 3.84 (s, 2H, CH_2_), 7.77–8.00 (m, 5H), 8.05 (s, 1H, NH), 11.35 (s, 1H, OH); ^13^C-NMR (DMSO) δ ppm: 41.34,127.31, 127.87, 128.00, 128.61, 132.89, 134.23, 167.87, 172.11; *m*/*z*: 179.06 (100.0%), 180.06 (10.3%), 181.06 (1.1%); Anal. Calcd. For C_9_H_9_NO_3_: C, 60.33; H, 5.06; N, 7.82; O, 26.79. Found: C, 60.35; H, 5.08; N, 7.80; O, 26.76.

*N*-((5-Amino-1,3,4-thiadiazol-2-yl)methyl) benzamide **5**. Yield: 92%; m.p.: 218–220 °C; IR (KBr ν_max_ in cm^−1^): 3360.45 (NH_2_), 3000 (C-H aromatic), 2970.76 (C=H), 1810.26 (C=O of amide), 1454.55 (CH of CH_2_); ^1^H-NMR (DMSO) δ ppm: 3.45 (s, 2H, CH_2_), 6.89 (s, 2H, NH_2_), 7.70–8.00 (m, 5H), 8.05 (s, 1H, NH); ^13^C-NMR (DMSO) δ ppm: 39.11,127.26, 127.69, 128.42, 128.76, 132.11, 134.21, 161.66, 167.89, 168.00; *m*/*z*: 234.06 (100.0%), 235.06 (11.8%), 236.05 (4.5%), 235.05 (1.5%); Anal. Calcd. For C_10_H_10_N_4_OS: C, 51.27; H, 4.30; N, 23.91; S, 13.69. Found: C, 51.29; H, 4.33; N, 23.89; S, 13.65.

(*E*)-*N*-((5-(4-Chlorobenzylideneamino)-1,3,4-thiadiazol-2-yl)methyl) benzamide **7a**. Yield: 95%; m.p.: 126–128 °C; IR (KBr ν_max_ in cm^−1^): 3355.41 (NH), 2970.76 (C=H), 1810.26 (C=O of amide), 1454.50 (CH of CH_2_); ^1^H-NMR (DMSO) δ ppm: 3.42 (s, 2H, CH_2_), 6.69–5.21 (m, 9H), 8.21 (s, 1H, NH), 10.33 (s, 1H, N=CH); ^13^C-NMR (DMSO) δ ppm: 39.11,127.36, 127.34, 127.51, 128.78, 128.98, 130.00, 130.63, 131.22, 133.23, 134.34, 134.51, 136.67, 160.00, 167.89, 168.00; *m*/*z*: 356.05 (100.0%), 358.05 (37.1%), 357.05 (20.7%), 359.05 (7.1%), 358.06 (1.6%), 360.04 (1.5%); Anal. Calcd. For C_17_H_13_ClN_4_OS: C, 57.22; H, 3.67; Cl, 9.94; N, 15.70; S, 8.99. Found: C, 57.24; H, 3.69; Cl, 9.97; N, 15.68; S, 8.98.

(*E*)-*N*-((5-(2,4-Dichlorobenzylideneamino)-1,3,4-thiadiazol-2-yl)methyl) benzamide **7b**. Yield: 92%; m.p.: 112–114 °C; IR (KBr ν_max_ in cm^−1^): 3352.41 (NH), 2975.76 (C=H), 1818.26 (C=O of amide), 1457.55 (CH of CH_2_); ^1^H-NMR (DMSO) δ ppm: 3.45 (s, 2H, CH_2_), 6.69–5.21 (m, 8H), 8.29 (s, 1H, NH), 10.33 (s, 1H, N=CH); ^13^C-NMR (DMSO) δ ppm: 40.11,127.36, 127.34, 127.51, 128.68, 128.98, 130.00, 130.53, 131.22, 133.13, 134.39, 134.41, 136.77, 161.00, 168.89, 169.00; *m*/*z*: 390.01 (100.0%), 392.01 (68.9%), 391.01 (20.7%), 393.01 (13.2%), 394.00 (13.1%), 395.01 (2.5%), 392.02 (1.8%), 394.01 (1.5%), 393.00 (1.0%); Anal. Calcd. For C_17_H_12_Cl_2_N_4_OS: C, 52.18; H, 3.09; Cl, 18.12; N, 14.32; S, 8.20. Found: C, 52.19; H, 3.11; Cl, 18.14; N, 14.30; S, 8.22.

(*E*)-*N*-((5-(4-Hydroxybenzylideneamino)-1,3,4-thiadiazol-2-yl)methyl) benzamide **7c.** Yield: 94%; m.p.: 118–120 °C; IR (KBr ν_max_ in cm^−1^): 3500.92 (OH), 3350.41 (NH), 2970.76 (C=H), 1810.26 (C=O of amide), 1458.50 (CH of CH_2_); ^1^H-NMR (DMSO) δ ppm: 3.44 (s, 2H, CH_2_), 5.43 (s,1H, OH), 6.69–5.20 (m, 9H), 8.21 (s, 1H, NH), 10.34 (s, 1H, N=CH); ^13^C-NMR (DMSO) δ ppm: 40.12,115.80, 116.19, 121.93, 125.20, 128.96, 129.41, 131.90, 134.60, 156.36, 163.36, 165.05, 168.54, 190.00; *m*/*z*: 338.08 (100.0%), 339.09 (18.6%), 340.08 (4.8%), 339.08 (2.3%), 340.09 (2.2%); Anal. Calcd. For C_17_H_14_N_4_O_2_S: C, 60.34; H, 4.17; N, 16.56; S 9.48. Found: C, 60.36; H, 4.19; N, 16.53; S, 9.45.

(*E*)-*N*-((5-(2-Hydroxybenzylideneamino)-1,3,4-thiadiazol-2-yl)methyl) benzamide **7d**. Yield: 94%; m.p.: 106–108 °C; IR (KBr ν_max_ in cm^−1^): 3500.90 (OH), 3350.58 (NH), 2970.66 (C=H), 1811.16 (C=O of amide), 1454.55 (CH of CH_2_); ^1^H-NMR (DMSO) δ ppm: 4.12 (s, 2H, CH_2_), 5.38 (s, 1H, OH), 7.02–7.71 (m, 4H), 7.81–8.10 (m, 5H), 8.16 (s, 1H, NH), 9.91 (s, 1H, N=CH); ^13^C-NMR (DMSO) δ ppm: 40.03,118.82, 120.52, 121.46, 126.89, 127.45, 127.91, 128.17, 129.80, 131.00, 132.41, 135.51, 160.55, 160.99, 169.09, 170.11; *m*/*z*: 338.08 (100.0%), 339.09 (18.6%), 340.08 (4.8%), 339.08 (2.3%), 340.09 (2.2%); Anal. Calcd. For C_17_H_14_N_4_O_2_S: C, 60.34; H, 4.17; N, 16.56; S, 9.48. Found: C, 60.36; H, 4.19; N, 16.53; S, 9.50.

(*E*)-*N*-((5-(4-Hydroxy-3-methoxybenzylideneamino)-1,3,4-thiadiazol-2-yl)methyl) benzamide **7e**. Yield: 92%; m.p.: 124–126 °C; IR (KBr ν_max_ in cm^−1^): 3500.90 (OH), 3350.31 (NH), 2971.76 (C=H), 1810.16 (C=O of amide), 1455.55 (CH of CH_2_); ^1^H-NMR (DMSO) δ ppm: 3.83 (s, 3H, OCH_3_), 4.10 (s, 2H, CH_2_), 5.35 (s, 1H, OH), 6.93 (d, *J* = 8.40 Hz,1H), 7.34 (d, *J* = 8.41 Hz,1H), 7.52 (s, 1H), 7.70–8.08 (m, 5H), 8.19 (s, 1H, NH), 10.00 (s, 1H, N=CH); ^13^C-NMR (DMSO) δ ppm: 39.87, 56.17, 113.17, 118.16, 122.15, 126.81, 127.01, 127.76, 128.28, 129.98, 130.16, 135.09, 149.71, 152.09, 159.99, 167.71, 169.18; *m*/*z*: 368.09 (100.0%), 369.10 (19.8%), 370.09 (4.8%), 370.10 (2.6%), 369.09 (2.3%); Anal. Calcd. For C_18_H_16_N_4_O_3_S: C, 58.68; H, 4.38;N, 15.21;S 8.70. Found: C, 58.70; H, 4.39; N, 15.19; S, 8.72.

(*E*)-*N*-((5-(3-Ethoxy-4-hydroxybenzylideneamino)-1,3,4-thiadiazol-2-yl)methyl) benzamide **7f**. Yield: 92%; m.p.: 130–132 °C; IR (KBr ν_max_ in cm^−1^): 3500.90 (OH), 3350.31 (NH), 2971.76 (C=H), 1810.16 (C=O of amide), 1457.55 (CH of CH_2_); ^1^H-NMR (DMSO) δ ppm: 1.32 (t, 3H, CH_3_), 4.09 (q, 2H, CH_2_), 4.46 (s, 2H, CH_2_), 5.35 (s, 1H, OH), 6.91–7.52 (m, 3H, CH), 7.63-8.09 (m, 5H, CH), 8.13 (s, 1H, NH), 10.00 (s, 1H, N=CH); ^13^C-NMR (DMSO) δ ppm: 14.87, 39.99, 64.57, 112.38, 116.59, 122.34, 127.00, 128.19, 128.88, 130.70, 132.55, 134.47, 148.83, 151.88, 161.66, 168.78; *m*/*z*: 382.11 (100.0%), 383.11 (22.9%), 384.11 (5.6%), 384.12 (2.1%); Anal. Calcd. For C_19_H_18_N_4_O_3_S: C, 59.67; H, 4.74; N, 14.65; S 8.38. Found: C, 59.69; H, 4.76; N, 14.61; S, 8.39.

(*E*)-*N*-((5-(4-Methoxybenzylideneamino)-1,3,4-thiadiazol-2-yl)methyl) benzamide **7g**. Yield: 95%; m.p.: 116–118 °C; IR (KBr ν_max_ in cm^−1^): 3352.18 (NH), 2971.66 (C=H), 1810.17 (C=O of amide), 1454.55 (CH of CH_2_); ^1^H-NMR (DMSO) δ ppm: 3.83 (s, 6H, OCH_3_), 4.19 (s, 2H, CH_2_), 7.06 (d, *J* = 8.40 Hz,1H), 7.16 (d, *J* = 8.41 Hz,1H), 7.60–8.06 (m, 7H), 8.17 (s, 1H, NH), 10.00 (s, 1H, N=CH); ^13^C-NMR (DMSO) δ ppm: 40.00, 55.89, 114.01, 114.45, 127.03, 127.52, 127.97, 128.01, 128.89, 130.71, 131.91, 132.96, 135.01, 161.00, 163.09, 169.99, 170.13; *m*/*z*: 352.10 (100.0%), 353.10 (21.8%), 354.10 (5.4%), 354.11 (1.8%); Anal. Calcd. For C_18_H_16_N_4_O_2_S: C, 61.35; H, 4.58; N, 15.90; S, 9.10. Found: C, 61.38; H, 4.59; N, 15.88; S, 9.13.

(*E*)-*N*-((5-(3,4-Dimethoxybenzylideneamino)-1,3,4-thiadiazol-2-yl)methyl) benzamide **7h**. Yield: 88%; m.p.: 120–122 °C; IR (KBr ν_max_ in cm^−1^): 3352.18 (NH), 2971.66 (C=H), 1810.17 (C=O of amide), 1456.75 (CH of CH_2_); ^1^H-NMR (DMSO) δ ppm: 3.83 (s, 6H, OCH_3_), 4.19 (s, 2H, CH_2_), 6.98–7.61 (m, 3H), 7.69–8.05 (m, 5H), 8.12 (s, 1H, NH), 9.98 (s, 1H, N=CH); ^13^C-NMR (DMSO) δ ppm: 39.96, 56.11, 108.92, 111.77, 124.69, 126.96, 127.58, 127.99, 128.81, 130.66, 132.10, 134.26, 149.92, 152.10, 159.91, 169.96, 170.09; *m*/*z*: 382.11 (100.0%), 383.11 (22.9%), 384.11 (5.6%), 384.12 (2.1%); Anal. Calcd. For C_19_H_18_N_4_O_3_S: C, 59.67; H, 4.74; N, 14.65; S 8.38. Found: C, 59.69; H, 4.76; N, 14.62; S, 8.36.

(*E*)-*N*-((5-(3,4,5-Trimethoxybenzylideneamino)-1,3,4-thiadiazol-2-yl)methyl) benzamide **7i**. Yield: 86%; m.p.: 138–140 °C; IR (KBr ν_max_ in cm^−1^): 3350.18 (NH), 2972.66 (C=H), 1810.17 (C=O of amide), 1456.87 (CH of CH_2_); ^1^H-NMR (DMSO) δ ppm: 3.85 (s, 9H, OCH_3_), 4.19 (s, 2H, CH_2_), 6.98–7.61 (m, 3H), 7.69–8.05 (m, 5H), 8.12 (s, 1H, NH), 9.98 (s, 1H, N=CH); ^13^C-NMR (DMSO) δ ppm: 39.96, 56.11, 108.92, 111.77, 124.69, 126.96, 127.58, 127.99, 128.81, 130.66, 132.10, 134.26, 149.92, 152.10, 159.91, 169.96, 170.09; *m*/*z*: 412.12 (100.0%), 413.12 (24.1%), 414.12 (5.9%), 414.13 (2.3%), 415.12 (1.1%); Anal. Calcd. For C_20_H_20_N_4_O_4_S: C, 58.24; H, 4.89; N, 13.58; S 7.77. Found: C, 58.27; H, 4.91; N, 13.54; S, 7.79.

(*E*)-*N*-((5-(2,4,5-Trimethoxybenzylideneamino)-1,3,4-thiadiazol-2-yl)methyl) benzamide **7j**. Yield: 88%; m.p.: 140–142 °C; IR (KBr ν_max_ in cm^−1^): 3350.18 (NH), 2972.66 (C=H), 1810.17 (C=O of amide), 1454.55 (CH of CH_2_); ^1^H-NMR (DMSO) δ ppm: 3.86 (s, 9H, OCH_3_), 4.20 (s, 2H, CH_2_), 6.99–7.65 (m, 3H), 7.67–8.07 (m, 5H), 8.17 (s, 1H, NH), 9.99 (s, 1H, N=CH); ^13^C-NMR (DMSO) δ ppm: 37.96, 55.35, 109.12, 110.97, 125.19, 126.96, 127.58, 128.99, 129.81, 131.66, 133.10, 135.26, 148.92, 153.10, 158.91, 168.96, 171.09; *m*/*z*: 412.12 (100.0%), 413.12 (24.1%), 414.12 (5.9%), 414.13 (2.3%), 415.12 (1.1%); Anal. Calcd. For C_20_H_20_N_4_O_4_S: C, 58.24; H, 4.89; N, 13.58; S 7.77. Found: C, 58.27; H, 4.92; N, 13.55; S, 7.78.

(*E*)-*N*-((5-(Furan-2-ylmethyleneamino)-1,3,4-thiadiazol-2-yl)methyl) benzamide **7k**. Yield: 85%; m.p.: 124–126 °C; IR (KBr ν_max_ in cm^−1^): 3350.41 (NH), 2970.76 (C=H), 1810.26 (C=O of amide), 1456.40 (CH of CH_2_); ^1^H-NMR (DMSO) δ ppm: 3.48 (s, 2H, CH_2_), 6.52 (t, 1H), 6.93 (d, *J* = 3.40 Hz,1H), 7.65 (d, *J* = 1.75 Hz, 1H), 7.80 (s, 1H, N=CH), 7.75–8.05 (m, 5H), 8.19 (s, 1H, NH); ^13^C-NMR (DMSO) δ ppm: 40.12, 112.26, 118.90, 126.99, 127.54, 128.04, 128.57, 131.11, 134.29, 144.48, 146.99, 150.44, 167.34, 168.09; *m*/*z*: 312.07 (100.0%), 313.07 (18.7%), 314.06 (4.5%), 314.07 (2.0%); Anal. Calcd. For C_15_H_12_N_4_O_2_S: C, 57.68; H, 3.87; N, 17.94; S, 10.27. Found: C, 57.69; H, 3.89; N, 17.92; S, 10.29.

(*E*)-*N*-((5-(Thiophen-2-ylmethyleneamino)-1,3,4-thiadiazol-2-yl)methyl) benzamide **7l**. Yield: 84%; m.p.: 130–132 °C; IR (KBr ν_max_ in cm^−1^): 3350.18 (NH), 2975.66 (C=H), 1815.17 (C=O of amide), 1455.67 (CH of CH_2_); ^1^H-NMR (DMSO) δ ppm: 4.46 (s, 2H, CH_2_), 7.17 (t, 1H, CH), 7.63–8.09 (m, 6H), 8.29 (s, 1H, NH); ^13^C-NMR (DMSO) δ ppm: 39.91, 127.00, 127.44, 127.99, 128.34, 128.89, 130.73, 132.77, 134.55, 142.98, 152.69, 167.98; *m*/*z*: 328.05 (100.0%), 329.05 (16.4%), 330.04 (9.1%), 329.04 (3.1%), 330.05 (2.0%), 331.04 (1.7%); Anal. Calcd. For C_15_H_12_N_4_O_2_S_2_: C, 54.86; H, 3.68; N, 17.06; S 19.53. Found: C, 54.88; H, 3.69; N, 17.03; S, 19.55.

### 3.2. In Vitro Anticancer Screening

The synthesized compounds **7**(**a**–**l**) were evaluated for their in vitro anticancer activity against MCF-7 (human breast cancer cell line), HeLa (human cervical cancer cell line), SKMEL-2 (human melanoma cancer cell line) and HL-60 (human leukemia cancer cell line) cancer cell lines. The GI_50_ values (concentration required to Growth inhibition of 50%) for the synthesized compounds were determined using the MTT assays method.

#### Experimental procedure for MTT assay

The stock solutions of test compounds were prepared in DMSO. After 24 h incubation, different concentrations (2, 4, 6, 8 µM) of compounds, made by serial dilution in culture medium, were added in 48 h incubation. The final concentration of DMSO was 0.01% in each well. A separate well containing 0.01% DMSO only was run as DMSO control, which was found inactive under applied conditions. The cell growth was determined using MTT (3-(4,5-dimethylthiazol-2-yl)-2,5-diphenyl tetrazolium bromide (Sigma, St. Louis, MO, USA) reduction assay, which is based on the ability of viable cells to reduce a soluble yellow tetrazolium salt to blue formazan crystal [38,39]. Briefly, after 48 h of treatments, the 10 µl of MTT dye, prepared in phosphate buffered saline (PBS) was added to all wells. The plates were then incubated for 4 h at 37 °C. Supernatant from each well was carefully removed, formazon crystals were dissolved in 100 µL of DMSO and absorbance at 540 nm wavelength was recorded and each concentration was tested in threefold. The GI_50_ values were determined as concentration of compounds that inhibited cancer cell growth by 50%.

### 3.3. Computational Study

#### Molecular Docking and In Silico ADMET Prediction

Molecular docking studies were performed in Maestro 9.1 using Glide v6.8 (Schrodinger, LLC, New York, NY, USA). All the compounds were built using Maestro build panels and optimized to lower energy conformers using Ligprep v3.5.9 (Schrodinger, LLC). The X-ray crystal structure of the Fibroblast stromelysin-1 enzyme (PDB code 1USN) [21] was obtained from the protein data bank and prepared for docking using protein preparation wizards [40]. After preparation, the hydrogen bond network in the enzyme structure was optimized using OPLS_2005 force field. The minimization was terminated when the energy converged or the route mean square deviation (RMSD) reached a maximum cutoff of 0.30 Å. Grids were then defined around refined and minimized structures by centering on ligand using default box size. The final docking studies for all the compounds were performed using the extra-precision (XP) docking mode on generated grid of protein structure [41]. The evaluation of ligand-protein binding was done with Glide (docking) score.

The ADMET properties of lower-energy conformers of all compounds were predicted using QikProp v3.5 (Schrödinger LLC).

## 4. Conclusions

In summary, a new scaffold of 12 *N*-((5-(substituted methylene amino)-1,3,4-thiadiazol-2-yl)methyl) benzamide derivatives **7**(**a**–**l**) has been synthesized in solvent free condition under microwave irradiation and evaluated for their potential in vitro anticancer activity on MCF-7, HeLa, SKMEL-2 and HL-60 human cancer cell lines by MTT assay using Adriamycin as a standard drug. The compounds **7k**, **7l**, **7b** and **7a** were found to be the most promising anticancer agents in this study. The compounds **7k**, **7l** and **7a** were found to be selective towards cancer cells, since they did not exhibit cytotoxicity even at GI_50_ > 100 μM on MCF-10A normal breast epithelial cell lines. The compound **7b** showed almost three times more selectivity for the MCF-7 cancer cell lines in comparison to the MCF-10A normal breast epithelial cell lines. 

The good docking scores as well as anticancer activity of these compounds have been justified by investigating their interaction at the active site of Fibroblast stromelysin-1, as found in the docking studies. The ADMET studies predicted good oral drug-like behavior of the newly synthesized derivatives. The studies presented here provide innovative agents for the development of novel anticancer agents targeting MMPs with the goal of improving cancer care.

## Figures and Tables

**Table 1:** Details of synthesis of *N*-((5-(Substituted methylene amino)-1,3,4-thiadiazol-2-yl)methyl) benzamide derivatives **7**(**a**–**l**) using conventional method and microwave method.

Entry			Conventional Method		Microwave Method	
	Solvent	Temperature °C	Time (h)	Yield (%)	Time (min)	Yield (%)
**7a**	Ethanol	80–90	3.30	78	8	95
**7b**	Ethanol	80–90	5.10	77	12	92
**7c**	Ethanol	80–90	5.00	78	12	94
**7d**	Ethanol	80–90	7.20	66	15	94
**7e**	Ethanol	80–90	7.25	52	15	92
**7f**	Ethanol	80–90	8.00	48	18	92
**7g**	Ethanol	80–90	5.25	76	12	95
**7h**	Ethanol	80–90	7.30	66	15	88
**7i**	Ethanol	80–90	8.00	68	20	86
**7j**	Ethanol	80–90	8.00	48	20	88
**7k**	Ethanol	80–90	4.15	44	10	85
**7l**	Ethanol	80–90	4.25	56	10	84

**Table 2:** In vitro anticancer activity of the synthesized compounds **7**(**a**–**l**).

Entry	GI_50_ µM				
	MCF-7	HeLa	SKMEL-2	HL-60	MCF-10A
**7a**	22.9	32.8	21.9	21.7	>100
**7b**	28.7	39.0	22.9	28.2	86.1
**7c**	32.4	41.1	27.5	33.3	ND
**7d**	36.7	52.4	34.0	40.2	ND
**7e**	35.2	46.8	28.1	39.6	ND
**7f**	38.4	49.2	30.0	37.5	ND
**7g**	41.0	66.1	46.4	42.4	ND
**7h**	46.2	71.7	49.1	48.2	ND
**7i**	49.0	78.0	52.6	45.8	ND
**7j**	51.4	78.8	55.7	49.9	ND
**7k**	11.7	23.8	19.6	35.5	> 100
**7l**	19.0	28.8	22.0	29.9	> 100
ADR	< 10	< 10	< 10	< 10	ND

ADR Adriamycin standard drug used.GI_50_ is the concentration exhibiting 50% inhibition of the growth, as compared to the growth of control.MCF-7 human breast cancer cell line, HeLa human cervical cancer cell line, SKMEL-2 human melanoma cancer cell line, HL-60 human leukemia cancer cell line, MCF-10A normal breast epithelial cell lines and ND = Not determined.

**Table 3:** Docking score of all the synthesized derivatives **7**(**a**–**l**).

Compound	Docking Score	Compound	Docking Score
**7a**	−6.61	**7h**	−6.33
**7b**	−6.56	**7i**	−6.61
**7c**	−5.93	**7j**	−5.10
**7d**	−5.82	**7k**	−7.56
**7e**	−5.84	**7l**	−7.32
**7f**	−5.66	ADR	−4.33
**7g**	−5.26		

**Table 4:** In silico physicochemical pharmacokinetic parameters important for good oral bioavailability of the synthesized compounds **7**(**a**–**l**).

Entry	M.W ^a^	LogP o/w ^b^ (−2.0–6.5)	*n*-ON ^c^ (<10)	*n*-OHNH ^d^ (<5)	PSA ^e^ (7–200)	log Khsa ^f^ (−1.5–1.2)	Log S ^g^ (−6–0.5)	% ABS ^h^	#Meta ^i^ (1–8)	Log HERG ^j^ Below−5	Lipinski Rule of 5 (≤1)
**7a**	356.8	4.82	5.5	1	76.2	0.31	−5.8	98	2	−6.8	0
**7b**	391.2	5.34	5.5	1	74.4	0.37	−6.1	99	2	−6.6	0
**7c**	338.3	5.18	6.2	2	98.4	0.02	−4.7	89	3	−6.7	0
**7d**	338.3	5.17	6	2	98.2	0.03	−4.3	88	3	−6.5	0
**7e**	368.4	5.27	7.2	1	133.3	−0.04	−4.7	75	3	−6.6	0
**7f**	382.4	5.09	7	2	105.7	0.16	−5.5	91	4	−6.9	0
**7g**	352.4	4.91	6.2	1	83.9	0.16	−5.1	100	3	−6.7	0
**7h**	382.4	5.20	7	1	89.0	0.19	−5.4	100	4	−6.7	0
**7i**	412.2	4.05	7.7	1	95.4	0.17	−5.4	100	5	−6.5	0
**7j**	412.2	4.08	7.7	1	97.6	0.19	−5.6	100	5	−6.6	0
**7k**	312.3	3.91	6	1	84.7	−0.11	−4.0	94	3	−6.3	0
**7l**	328.4	4.11	5	1	85.6	−0.21	−4.2	95	3	−6.2	0

^a^ Molecular weight of the molecule; ^b^ Predicted octanol-water partition coefficient (log Po/w) (−2.0 to 6.5); ^c^ *n*-ON number of hydrogen bond acceptors ≤10; ^d^ *n*-OHNH number of hydrogen bonds donors ≤5; ^e^ Polar surface area (PSA) (7.0–200.0); ^f^ Logarithm of predicted binding constant to human serum albumin (log Khsa) (−1.5 to 1.2); ^g^ Logarithm of aqueous solubility(Log S) (−6 to 0.5); ^h^ Percentage of human oral absorption (% ABS) (>80% is high, <25% is poor); #Metabolism ^I^ (1–8); ^j^ Predicted IC_50_ value for blockage of HERG K^+^ channels (log Khsa) (−1.5 to 1.2).
